# dsRNA: mechanisms of gene expression regulation, disease pathogenesis, and therapeutic potential

**DOI:** 10.1080/15476286.2026.2745886

**Published:** 2026-11-03

**Authors:** Shuiping Liu, Chao Zhe, Jiyun Hou, Yuling Fang, Haiying Yang, Yaoxin Chen, Shunhong Wu, Qin Xiang, Yuqian Zhang, Jiawen Fang, Yuanyuan Zhou, Linhai Li, Bin Xiao

**Affiliations:** a School of Public Health, Dali University, Dali, Yunnan, China; b Department of Laboratory Medicine, The Affiliated Qingyuan Hospital (Qingyuan People’s Hospital), Guangzhou Medical University, Qingyuan, China; c School of Clinical Medicine, Guangzhou Medical University, Guangzhou, China; d Graduate School, Guangzhou University of Chinese Medicine, Guangzhou, China; e Department of Laboratory Medicine, Southern Medical University Hospital of Integrated Traditional Chinese and Western Medicine, Southern Medical University, Guangzhou, China

**Keywords:** Double-stranded RNA, gene expression regulation, dsRNA-binding proteins, RNA–RNA interactions, dsRNA-based therapeutics

## Abstract

Double-stranded RNA (dsRNA) has long been recognized as a viral pathogen-associated molecular pattern that activates innate immunity. However, dsRNA is increasingly recognized as a regulatory RNA species involved in RNA homeostasis, gene expression, and cell fate determination. In this review, we discuss canonical dsRNA-dependent mechanisms, including RNA interference (RNAi), with particular emphasis on nuclear RNAi, and dsRNA-sensing networks, together with broader RNA–RNA duplex-associated regulatory processes. We also consider structurally distinct RNA–DNA hybrids that functionally converge with dsRNA-responsive innate immune pathways. We further examine how dysregulation of dsRNA production, processing, sensing, and clearance contributes to cancer, autoimmune disorders, and neurodegenerative diseases, and how these mechanisms can be therapeutically exploited. Collectively, these insights establish a conceptual framework for understanding the roles of dsRNA in physiological regulation, disease pathogenesis, and therapeutic applications.

## GRAPHICAL ABSTRACT

**Figure:**
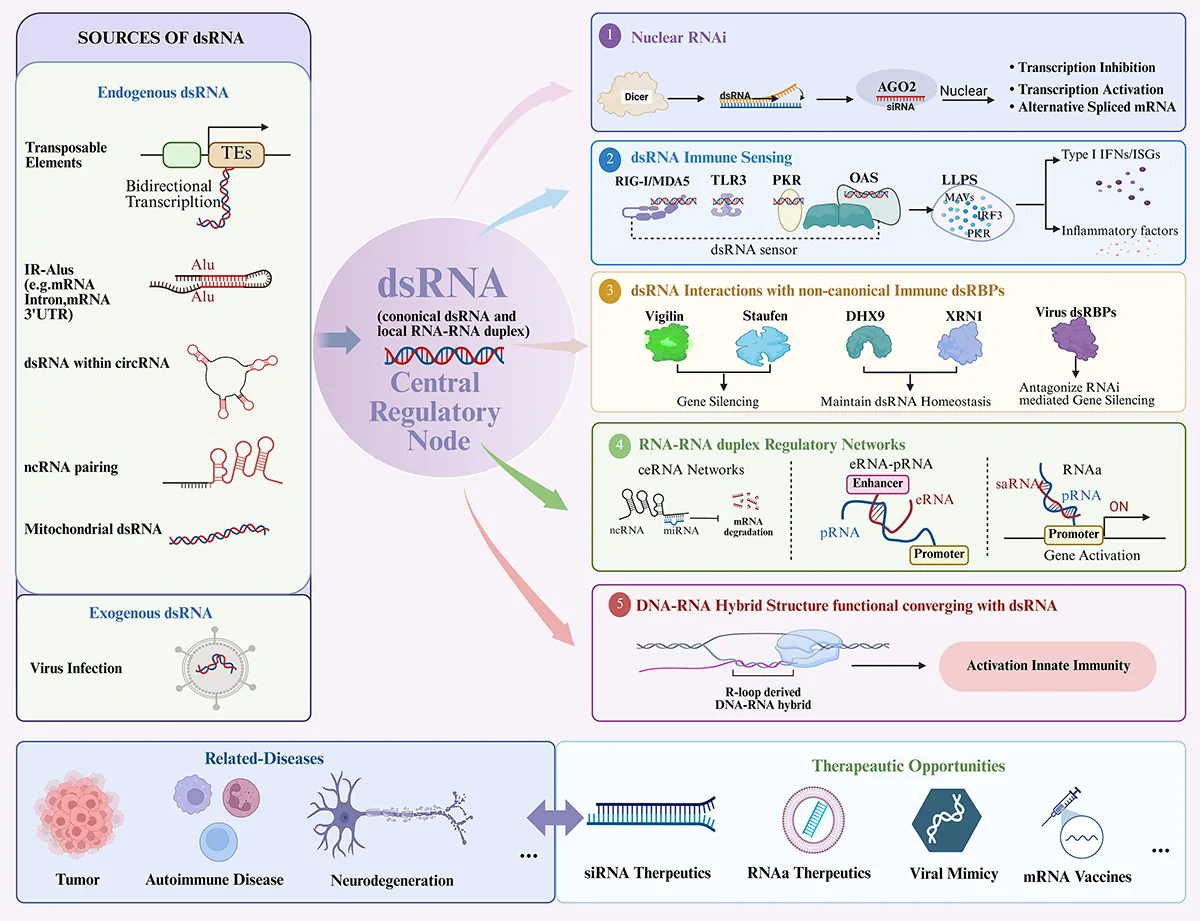

## 1. Introduction

Double-stranded RNA (dsRNA) consists of two complementary RNA strands stabilized by Watson–Crick base pairing and typically adopts a characteristic A-form double-helical conformation. Initially, dsRNA was primarily recognized as a pathogen-associated molecular pattern (PAMP) closely linked to viral infections [1]. However, accumulating evidence has demonstrated that endogenous dsRNA can also be widely generated under diverse physiological and pathological conditions and may abnormally accumulate during cellular stress or disease states. These dsRNAs are no longer considered merely transcriptional by-products but rather function as critical regulatory elements involved in multiple biological processes, including antiviral immune responses, tumour immunity, RNA homoeostasis and cell fate determination [2,3]. Consistently, dysregulation of dsRNA metabolism or aberrant sensing has been closely implicated in the pathogenesis of multiple human diseases, including rare disorders such as Aicardi–Goutières syndrome (AGS) and Singleton–Merten syndrome (SMS) [4,5]. Moreover, as our understanding of the structural diversity and regulatory functions of RNA has deepened, some structurally distinct local RNA–RNA duplexes have been found to functionally converge with canonical dsRNA-dependent pathways. These local RNA–RNA duplexes can arise from intramolecular base pairing within individual RNA molecules or from intermolecular pairing between distinct RNAs [6]. In this review, we discuss such structures only where they functionally converge with canonical dsRNA-dependent pathways. Although this field is advancing rapidly, the mechanisms underlying dsRNA generation, specific recognition, and the mediation of distinct functional outputs, as well as their roles in disease development and the therapeutic strategies based on dsRNA regulation, remain to be systematically integrated. Accordingly, we systematically summarize the endogenous origins and structural characteristics of dsRNA, with particular emphasis on canonical dsRNA-dependent mechanisms, including RNAi, innate immune sensing, and gene expression regulation mediated by non-canonical dsRNA-binding proteins, while further considering regulatory processes involving local RNA–RNA duplexes. We additionally discuss RNA–DNA hybrids derived from R-loops, which functionally converge with dsRNA-responsive innate immune pathways. On this basis, we further discuss the functional significance of dsRNA in cancer, autoimmune diseases, and neurodegenerative diseases, as well as therapeutic strategies based on dsRNA regulation, their current challenges, and future perspectives.

## 2. Endogenous sources and biogenesis of dsRNA

### 2.1. Epigenetic and epitranscriptomic dysregulation in dsRNA biogenesis

#### 2.1.1. Epigenetic dysregulation of transposable elements as a source of dsRNA

Epigenetic dysregulation–induced activation of transposable elements (TEs) represents one of the major sources of endogenous dsRNA. Under physiological conditions, TEs are largely silenced through DNA methylation-mediated epigenetic repression. However, loss or reduction of DNA methylation can derepress specific TE classes, particularly long interspersed nuclear elements (LINEs) and endogenous retroviruses (ERVs), leading to the production of complementary sense and antisense RNAs. These transcripts may arise from separate transcriptional units rather than from a single bidirectional promoter, and can anneal through overlapping complementary regions to form dsRNA. In contrast, short interspersed nuclear elements (SINEs) primarily generate immunogenic dsRNA through inverted Alu repeats (IR-Alus), which undergo intramolecular base-pairing within the same transcripts to form stable stem-loop structures [7,8] (Figure 1(A)). It is noteworthy that the contribution of repetitive elements to endogenous dsRNA formation is highly dependent on species-specific genomic landscapes. Alu elements, a primate-specific family of SINEs, constitute more than 10% of the human genome and represent a major source of endogenous immunogenic dsRNA. However, Alu elements are not conserved across other mammalian lineages, which may utilize alternative repetitive elements or genomic configurations to generate RNA duplex structures. For instance, rodents lack Alu elements but contain independently expanded SINE families, including B1 and B2 elements, which can produce structurally analogous dsRNA substrates despite their distinct evolutionary origins [9].

**Figure 1.**
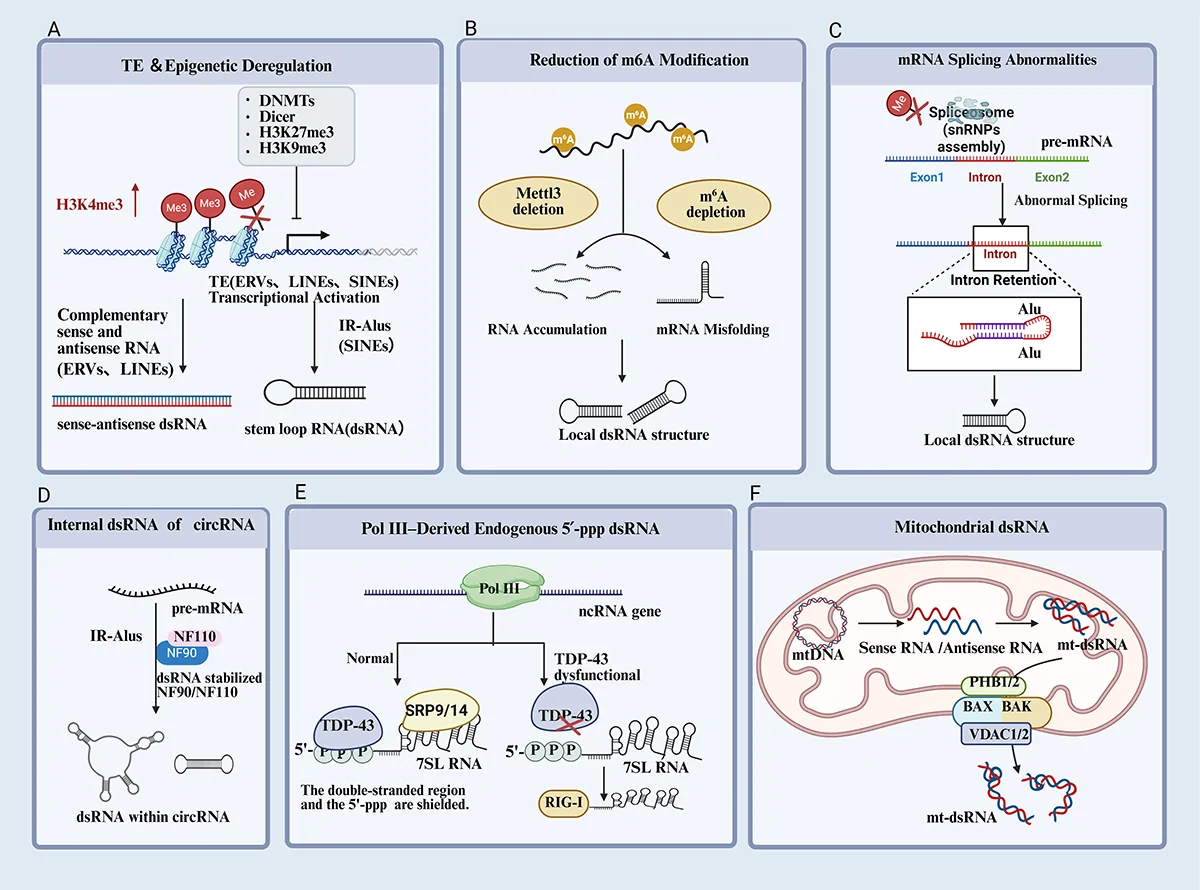
Major endogenous sources and biogenesis pathways of dsRNA. (A) Epigenetic dysregulation activates TEs, promoting the production of complementary sense and antisense RNA that anneal to form dsRNA. (B) epitranscriptomic dysregulation through loss of m6A modification promotes dsRNA accumulation. (C) aberrant mRNA splicing and intron retention–mediated dsRNA formation. (D) intramolecular dsRNA structures within circRNAs. (E) RNA polymerase-III derived transcripts as endogenous sources of 5′-ppp dsRNA. (F) mitochondrial dsRNA biogenesis and cytosolic release. Created in https://BioRender.com.

RNAi provides an additional layer of epigenetic control over the activity of TEs by promoting DNA methylation-dependent silencing. The RNase III enzyme Dicer processes TE-derived dsRNA into small interfering RNAs (siRNAs), which in turn recruit DNA methyltransferases (DNMTs) to establish and maintain DNA methylation–dependent TE silencing. When Dicer function is impaired, siRNA biogenesis is compromised, leading to reduced methylation and transcriptional upregulation of transposable elements such as long interspersed nuclear element-1 (LINE-1) [10]. LINE-1 represents a more specific locus-dependent case. Its 5′ untranslated region (UTR) contains both sense and antisense promoter activities, which can generate partially complementary transcripts and anneal to form dsRNA structures [10].

Aberrant histone modifications also influence the activation of TEs, thereby regulating dsRNA formation. Repressive histone marks, such as histone H3 lysine 9 trimethylation (H3K9me3) and histone H3 lysine 27 trimethylation (H3K27me3), are essential for maintaining condensed heterochromatin, thereby suppressing the transcription of TEs, including LINE-1 and ERVs, and consequently limiting dsRNA production. Conversely, activating histone marks, such as histone H3 lysine 4 dimethylation (H3K4me2) and histone H3 lysine 4 trimethylation (H3K4me3), promote chromatin accessibility and enhance the transcription of ERVs and IR-Alus within the SINE family, ultimately driving dsRNA biogenesis (Figure 1(A)). Notably, selective removal of these activating marks by lysine-specific demethylase 1 (LSD1) maintains TEs in a silenced state, thereby suppressing dsRNA generation [11,12].

#### 2.1.2. dsRNA formation induced by m^6^A loss and epitranscriptomic dysregulation

Current evidence indicates that reduced N^6^-methyladenosine (m^6^A) modification significantly facilitates the formation and accumulation of endogenous dsRNA [13]. Mechanistically, m^6^A modification introduces a methyl group at the N^6^ position of adenosine, weakening A–U base-pairing affinity and destabilizing RNA duplexes, ultimately inhibiting dsRNA formation [14,15]. Conversely, upon m^6^A depletion, this inhibition is relieved, resulting in the aberrant accumulation of RNA transcripts that would normally be recognized and degraded by m^6^A reader proteins such as YTH domain-containing family protein 2 (YTHDF2) [16], particularly transcripts originating from intronic regions and 3′ UTRs. Because these transcripts are enriched in TE-derived sequences and inverted repeats (e.g. Alu elements), they are more prone to forming stable dsRNA structures through complementary base-pairing [17,18] (Figure 1(B)).

### 2.2. dsRNA formation induced by aberrant mRNA splicing

Impaired mRNA splicing and the resulting accumulation of aberrantly retained introns provide a structural basis for the formation of endogenous dsRNA. Under pharmacological perturbation, exemplified by treatment with the type I protein arginine methyltransferase (PRMT) inhibitor MS023, reduced PRMT1 activity leads to insufficient arginine methylation of multiple splicing factors, including serine/arginine-rich (SR) proteins and small nuclear ribonucleoprotein (snRNP)-associated proteins. This modification defect weakens the interactions among splicing factors, their cognate RNA substrates, and core spliceosomal components, thereby triggering widespread intron retention. Notably, retained intronic sequences enriched in IR-Alus readily undergo complementary base-pairing, ultimately facilitating the formation of stable dsRNA structures [19] (Figure 1(C)).

Based on the mechanism by which aberrant mRNA splicing promotes dsRNA formation, spliceosome-targeted therapy (STT) has emerged as a promising strategy for cancer immunotherapy. For instance, in triple-negative breast cancer, spliceosome inhibitors targeting splicing factor 3B subunit 1 (SF3B1) disrupt spliceosomal function, leading to widespread intron retention and promoting dsRNA formation. The aberrantly accumulated dsRNA subsequently activates dsRNA sensors, such as RIG-I-like receptors (RLRs), which in turn activate the expression of type I interferons (IFN-I) and inflammatory genes. Collectively, these events reshape the immune microenvironment and potentiate antitumor immune responses [20].

### 2.3. Intramolecular dsRNA structures within circular RNA

Circular RNAs (circRNAs) are covalently closed RNA molecules generated from pre-mRNA through back-splicing [21]. In most circRNAs, intronic IR-Alus facilitate the formation of stable, albeit partially imperfect, dsRNA structures that typically span 16–26 nucleotides (nt) [22] (Figure 1(D)). During this process, the back-splicing factors NF90 and NF110 not only promote transcript circularization but also directly bind to dsRNA structures to reinforce RNA duplex stability [23]. This mechanism highlights the structural and mechanistic diversity underlying endogenous dsRNA biogenesis.

Under homoeostatic conditions, dsRNA regions within circRNAs serve as high-affinity binding platforms for the double-stranded RNA-binding domains (dsRBDs) of protein kinase R (PKR), preserving immune homoeostasis [22]. However, upon viral infection, these internal dsRNA structures are degraded by RNase L, resulting in the release of PKR from its inhibitory circRNA-bound state and subsequent activation of the PKR-mediated immune signalling pathway.

### 2.4. RNA polymerase III–transcribed RNAs as endogenous sources of 5′-ppp dsRNA

RNA polymerase III (Pol III) is the primary enzyme responsible for transcribing a diverse set of short non-coding RNAs (ncRNAs) in eukaryotes, including transfer RNA (tRNA), 5S ribosomal RNA (5S rRNA), 7SK small nuclear RNA (7SK snRNA), 7SL signal recognition particle RNA (7SL RNA), and Alu RNA [24]. Among these transcripts, 7SL RNA and Alu RNA contain extensive double-stranded regions and retain 5′-triphosphate (5′-ppp) termini, rendering them potential endogenous ligands for dsRNA-sensing pathways. Under physiological conditions, these double-stranded regions are masked by the SRP9/14 complex, whereas the 5′-ppp termini are shielded by TAR DNA-binding protein 43 (TDP-43) [25] (Figure 1(E)). When TDP-43 function is compromised, 7SL RNA and Alu RNA accumulate as immunostimulatory dsRNA, accompanied by exposure of their 5′-ppp termini, thereby enhancing their immunogenic potential [26]. Harnessing this regulatory axis, recent studies have shown that viral delivery of 7SL RNA into chimeric antigen receptor (CAR) T cells promotes their expansion and differentiation towards an effector memory phenotype while simultaneously suppressing T-cell exhaustion. This strategy ultimately improves antitumor efficacy against solid tumours and prolongs therapeutic durability [27].

### 2.5. Mitochondrial dsRNA biogenesis

Mitochondrial DNA (mtDNA) is a circular double-stranded genome that undergoes bidirectional transcription from promoters on both the heavy (H) and light (L) strands. This process generates complementary RNA transcripts that subsequently anneal to form mitochondrial dsRNA (mt-dsRNA) (Figure 1(F)). Under physiological conditions, mt-dsRNA abundance is tightly regulated by a surveillance complex composed of polynucleotide phosphorylase (PNPase), the RNA helicase SUV3, and mitochondrial poly(A) polymerase (MTPAP). Together, these factors maintain low steady-state levels of light-strand–derived antisense RNA, thereby limiting dsRNA formation [28]. In addition, dysregulation of RNA-processing factors, including G-rich RNA sequence-binding factor 1 (GRSF1) and mitochondrial single-stranded DNA-binding protein (mtSSB), as well as defects in 5-methylcytosine (m^5^C) RNA modification [29–31], has been implicated in the regulation of mt-dsRNA biogenesis. Moreover, extrinsic stressors such as hypoxia and ionizing radiation can further modulate mt-dsRNA production [32]. When mt-dsRNA accumulates beyond a critical threshold, it can be released into the cytosol through pores formed by outer mitochondrial membrane proteins, including voltage-dependent anion channel 1/2 (VDAC1/2), BAX/BAK complexes, and inner membrane proteins prohibitin 1/2 (PHB1/2). Once in the cytoplasm, mt-dsRNA acts as a novel damage-associated molecular pattern (DAMP) and is recognized by cytosolic dsRNA sensors (e.g. RLRs). This recognition triggers IFN-I production and participates in innate immune defence [33].

## 3. Structural features and recognition principles of dsRNA

### 3.1. Core structural features of dsRNA

The canonical structure of dsRNA adopts an A-form helical conformation characterized by a wide, shallow minor groove and a narrow, deep major groove. The expanded minor groove provides the essential structural platform for protein recognition. Another defining feature is the continuous presence of 2′-hydroxyl (2′-OH) groups on the ribose within the minor groove, which establish a characteristic hydration and hydrogen-bonding network. This network contributes to the stabilization of the A-form helix and enhances the thermal stability of dsRNA [34]. Furthermore, the A-form conformation of dsRNA is not a static structure but exhibits substantial structural plasticity. Changes in ionic conditions and temperature can alter the major groove width and helical twist angles, thereby influencing the structural accessibility of dsRNA [35].

Within cells, dsRNA exhibits considerable diversity in both length and structural contexts. Canonical long dsRNAs include viral replication intermediates and extended duplexes formed by complementary sense and antisense transcripts, whereas short dsRNAs include siRNAs of approximately 21–25 nt generated by Dicer-mediated cleavage of longer dsRNA precursors. The length is an important determinant of selective recognition by dsRNA-binding receptors and proteins. For example, the dsRNA sensor melanoma differentiation-associated protein 5 (MDA5) preferentially recognizes long dsRNA species exceeding approximately 500 bp, whereas PKR can bind dsRNA of at least 30 bp [36,37]. Beyond these canonical dsRNA species, local RNA–RNA duplexes can be formed through intramolecular base pairing, such as imperfect duplex regions within circRNAs and IR-Alu-containing transcripts, or through intermolecular pairing between distinct RNA molecules [6–8]. These local duplexes can also serve as direct substrates for dsRBPs, providing an important structural basis for the broader discussion of dsRNA-associated regulatory mechanisms below.

### 3.2. Structure-specific recognition by dsRNA–binding proteins

Owing to its A-form helical architecture, the narrow and deep major groove of dsRNA limits direct sequence-specific base recognition. Consequently, most double-stranded RNA-binding proteins (dsRBPs) primarily rely on their dsRBDs to recognize structural features rather than specific sequences. Mechanistically, regions 1 and 2 of the dsRBD typically engage the 2′-OH groups within the minor groove, whereas region 3 interacts with the phosphodiester backbone. Together, these interactions confer a high degree of structure-dependent binding specificity [38]. This recognition mode is exemplified by key dsRBPs, such as Dicer, Argonaute 2 (AGO2), and the cofactor TAR RNA-binding protein (TRBP) [39]. Importantly, sequence-specific recognition is not entirely absent. Emerging evidence indicates that Dicer can specifically recognize a GYM motif (G–pyrimidine–mismatched base) located near the cleavage site within the double-stranded region of pre-miRNA, thereby enhancing cleavage precision and efficiency [40].

Local structural features of dsRNA likewise play critical roles in modulating interactions with dsRBPs. The termini of dsRNA can exist as either blunt ends or characteristic 2-nt 3′ overhangs, and these distinct end geometries influence the anchoring and positioning of dsRBPs. For example, Dicer utilizes the characteristic 2-nt 3′ overhang to accurately position and process dsRNA substrates [41]. In addition, non-canonical structural elements, including mismatches, bulges, and hairpins, can alter the width of the major groove and reshape the protein recognition surface. These perturbations can either augment or attenuate binding affinity for various dsRBPs [42]. Ultimately, these structural variations direct dsRNA into distinct functional pathways, such as triggering RNAi or activating innate immune responses.

### 3.3. Structural modifications of dsRNA in self/non-self discrimination

Intracellular dsRNA can harbour diverse structural modifications that facilitate discrimination between self and non-self. These modifications primarily include adenosine-to-inosine (A-to-I) editing mediated by adenosine deaminase acting on RNA 1 (ADAR1), 5′-terminal phosphate modifications such as 5′-ppp and 5′-diphosphate (5′-pp), and ribose-2′-O-methylation (2′-O-Me). These modifications influence whether dsRNA is recognized as self or non-self, thereby determining its participation in gene regulation. A-to-I editing serves as a molecular tag of host-derived self dsRNA, effectively preventing its recognition by dsRNA sensors [43]. Conversely, 5′-ppp and 5′-pp termini are characteristic features of certain viral dsRNA species, facilitating their recognition by host sensors as non-self danger signals, and subsequently triggering antiviral immune responses. To avoid aberrant recognition, endogenous host dsRNA undergoes processing events such as capping and dephosphorylation, which minimize exposure of 5′-ppp moieties and thereby prevent inappropriate immune activation [44,45].

Ribose-2′-O-methylation, which involves the addition of a methyl group to the ribose 2′-OH position, can attenuate host recognition of dsRNA. This modification suppresses activation of dsRNA sensors and subsequently inhibits downstream gene regulatory signalling. A representative example is the Ebola virus (EBOV) L protein, which possesses intrinsic 2’-O-methyltransferase activity. This enzyme introduces a 2’-O-Me modification at the first nucleotide of the viral dsRNA 5’ terminus, thereby causing host sensors to interpret viral dsRNA as self and facilitating immune evasion [46]. Collectively, these modifications influence dsRNA recognition and downstream gene regulatory signalling, thereby helping cells balance endogenous RNA tolerance with antiviral immune surveillance.

## 4. dsRNA-mediated mechanisms of gene expression regulation

### 4.1. dsRNA-mediated regulation of gene expression through RNA interference

RNAi is a highly conserved gene-silencing mechanism initiated by dsRNA. The canonical RNAi pathway relies on Dicer to process long dsRNA into siRNAs of approximately 21–25 nt. These siRNAs are subsequently loaded onto AGO2 to form the RNA-induced silencing complex (RISC), which mediates sequence-specific degradation of complementary mRNA targets [41] (Figure 2(A)). Although this mechanism has been extensively validated, this section focuses on the broader roles of dsRNA-mediated RNAi in regulating gene expression beyond this canonical post-transcriptional silencing.

**Figure 2.**
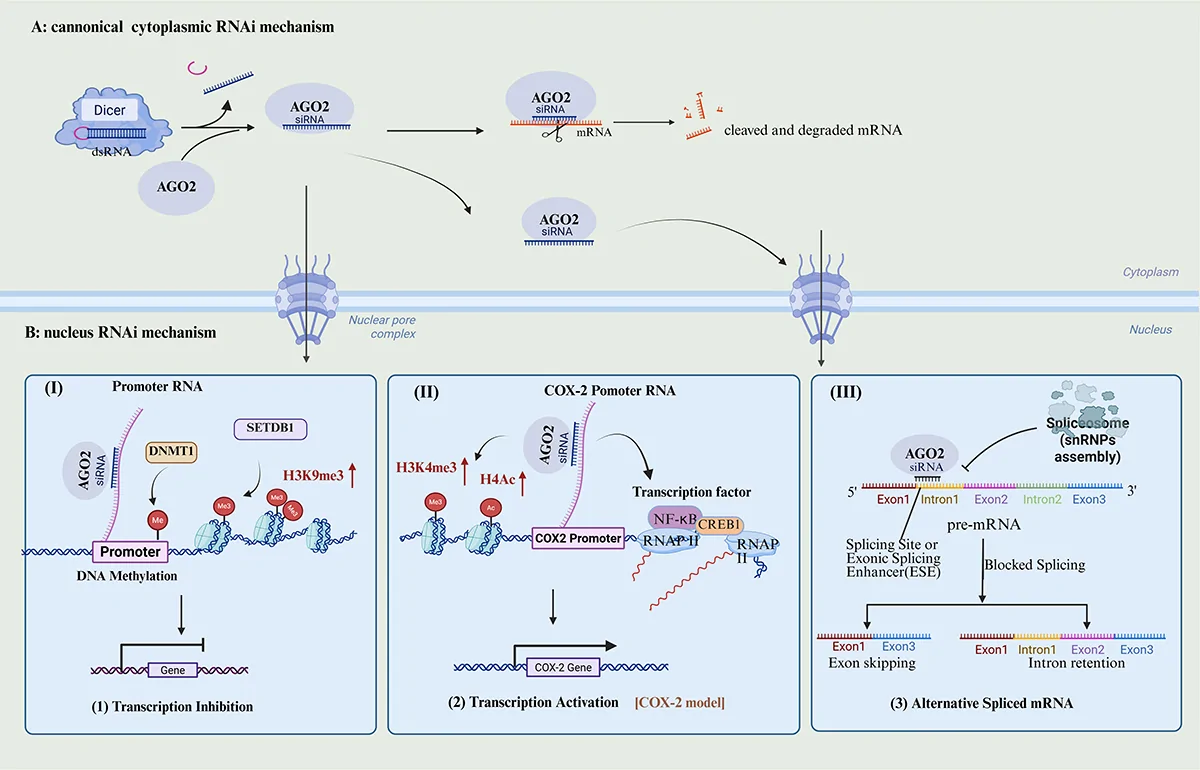
dsRNA-mediated regulation of gene expression through RNAi. (A) Canonical cytoplasmic RNAi pathway. (B) Nuclear RNAi–mediated regulation, including (I) transcriptional silencing, (ii) transcriptional activation, and (iii) alternative splicing regulation. Created in https://BioRender.com.

Several studies have reported that core RNAi factors, including siRNAs, AGO2, and Dicer, are also present in the nuclei of mammalian cells [47–49], challenging the long-held view that key RNAi factors are confined to the cytoplasm [50]. In specific experimental systems, nuclear siRNA-AGO2 complexes appear to recognize promoter- or chromatin-associated non-coding RNAs. For example, targeting promoter-associated ncRNAs has been associated with the recruitment of DNA methyltransferase 1 (DNMT1) and the induction of promoter DNA methylation. It has also been linked to the recruitment of histone methyltransferases such as SET domain bifurcated histone lysine methyltransferase 1 (SETDB1) and H3K9me3 enrichment at specific genomic loci, thereby facilitating heterochromatin formation and transcriptional silencing of nuclear genes [51] (Figure 2, B(I)). siRNA-AGO2 complexes can also target chromatin-associated RNAs located distal to promoters. For example, members of the very long intergenic non-coding RNA (vlincRNA) family, exemplified by VINK, can similarly induce transcriptional silencing at their corresponding promoters [49]. However, nuclear RNAi effects are not restricted to transcriptional silencing. In the cyclooxygenase-2 (COX-2) model, the siRNA-AGO2 complex binds to promoter-associated COX-2 RNA and promotes recruitment of RNA pol II, together with transcription factors such as NF-κB and CREB1. This process is accompanied by the enrichment of activating histone marks, including H3K4me3 and histone H4 acetylation (H4Ac), thereby promoting COX-2 transcriptional activation [52] (Figure 2, B(II)). These findings suggest that nuclear RNAi may exert dual effects on transcription. Overall, these studies demonstrate that nuclear RNAi effects can be experimentally induced, but also indicate that their outcomes are dependent on the genomic locus, cell type, and triggering conditions rather than representing a uniform mode of transcriptional regulation.

Beyond transcriptional regulation, siRNA can target critical intron–exon junctions, thereby interfering with the recruitment of splicing factors and modulating pre-mRNA splicing, ultimately generating distinct mRNA isoforms [53] (Figure 2, B(III)). RNAi and mRNA splicing may exhibit reciprocal interplay. Fuchs and colleagues demonstrated that different mRNA splice variants display differential sensitivity to RNAi-mediated silencing. In particular, intron-retaining mRNA isoforms can evade RNAi-dependent degradation, indicating that alternative splicing patterns can influence the efficiency of siRNA-mediated gene silencing [54].

Despite these findings, the physiological significance of nuclear RNAi in regulating endogenous gene expression in mammalian cells remains an open question. Compared with plants and fungi, where endogenous siRNA pathways derived from dsRNA have been extensively demonstrated to direct DNA methylation and heterochromatin formation [55,56], the existence and physiological relevance of endogenous nuclear RNAi mechanisms in mammals remain insufficiently defined. Although endogenous siRNA populations have been detected in mammalian cells, their abundance, diversity, and contribution to chromatin regulation seem to be more limited and highly dependent on cellular and genomic context. Moreover, many reported nuclear RNAi effects are based on exogenous siRNA systems or specific experimental models [49,50], making it difficult to determine their broader physiological relevance. In the future, studies using endogenous dsRNA substrates, physiologically relevant models, and genome-wide approaches will be required to clarify the prevalence and biological significance of nuclear RNAi-mediated regulation in mammals.

### 4.2. dsRNA sensing in innate immune gene expression

The innate immune system initiates host defence responses through the recognition of microbial pathogens and other danger signals. As a prototypical PAMP, dsRNA is recognized by multiple innate immune sensor systems. In the cytoplasm, dsRNA is primarily sensed by RLRs, PKR, and 2’−5’-oligoadenylate synthetases (OASes), whereas endosomal dsRNA is predominantly recognized by Toll-like receptor 3 (TLR3), collectively initiating innate immune response cascades [57,58]. Activation of these sensor pathways reshapes cellular gene expression programmes through multiple mechanisms, including transcriptional induction of interferon-stimulated genes (ISGs), widespread translational inhibition, and targeted RNA degradation [59].

#### 4.2.1. dsRNA-induced interferon gene transcription through RLRs and TLR3

RLRs and TLR3 constitute the two principal pathways through which cells sense dsRNA and induce interferon gene expression, representing cytosolic and endosomal surveillance mechanisms, respectively.

The human RLR family comprises retinoic acid-inducible gene I (RIG-I), MDA5, and laboratory of genetics and physiology 2 (LGP2), of which RIG-I and MDA5 serve as the primary cytosolic dsRNA sensors. RIG-I preferentially recognizes short viral dsRNA bearing 5′-ppp or 5′-pp termini [57,58]. These structural features are typically eliminated during the maturation processing of endogenous host RNA. Although RIG-I can bind endogenous dsRNA, its caspase activation and recruitment domains (CARDs) are only transiently exposed and are rapidly re-sequestered by the Hel2i domain. This mechanism prevents sustained signalling and avoids inappropriate activation by host self-derived dsRNA [59]. In contrast, MDA5 exhibits a strong preference for long dsRNA duplexes in a length-dependent manner. Moreover, ATP hydrolysis enables MDA5 to dissociate from short endogenous dsRNA, thereby enhancing its selectivity for the long and stable dsRNA structures commonly associated with viral replication [36]. Upon successful dsRNA recognition, RIG-I and MDA5 undergo conformational changes that expose their N-terminal CARDs. This exposure facilitates the activation and subsequent prion-like polymerization of the mitochondrial antiviral-signalling protein (MAVS). Activated MAVS serves as a signalling platform for the activation of the interferon regulatory factors 3 and 7 (IRF3/7) and nuclear factor-κB (NF-κB). These transcription factors collectively induce robust transcription of IFN-I, particularly IFN-β, together with a broad repertoire of ISGs (e.g. ISG15, MX1, RSAD2) (Figure 3), thereby establishing an antiviral state [60,61]. Notably, the immunogenicity of dsRNA is determined not only by its length and terminal modifications but also by its higher-order conformational states. Under certain stress conditions, a subset of dsRNA adopts a left-handed helical structure known as Z-RNA, which is recognized by the cytosolic Z-nucleic acid sensor Z-DNA binding protein 1 (ZBP1) through its Zα2 domain. This interaction activates the MAVS–TBK1 (TANK-binding kinase 1)–IRF3 signalling axis, triggering the induction of IFN-I and ISGs [62]. In addition, LGP2 functions as an important positive regulator of RLR signalling. Although it lacks CARDs, LGP2 facilitates the assembly and signalling activity of RIG-I/MDA5–dsRNA complexes, thereby amplifying antiviral responses [63,64].

**Figure 3.**
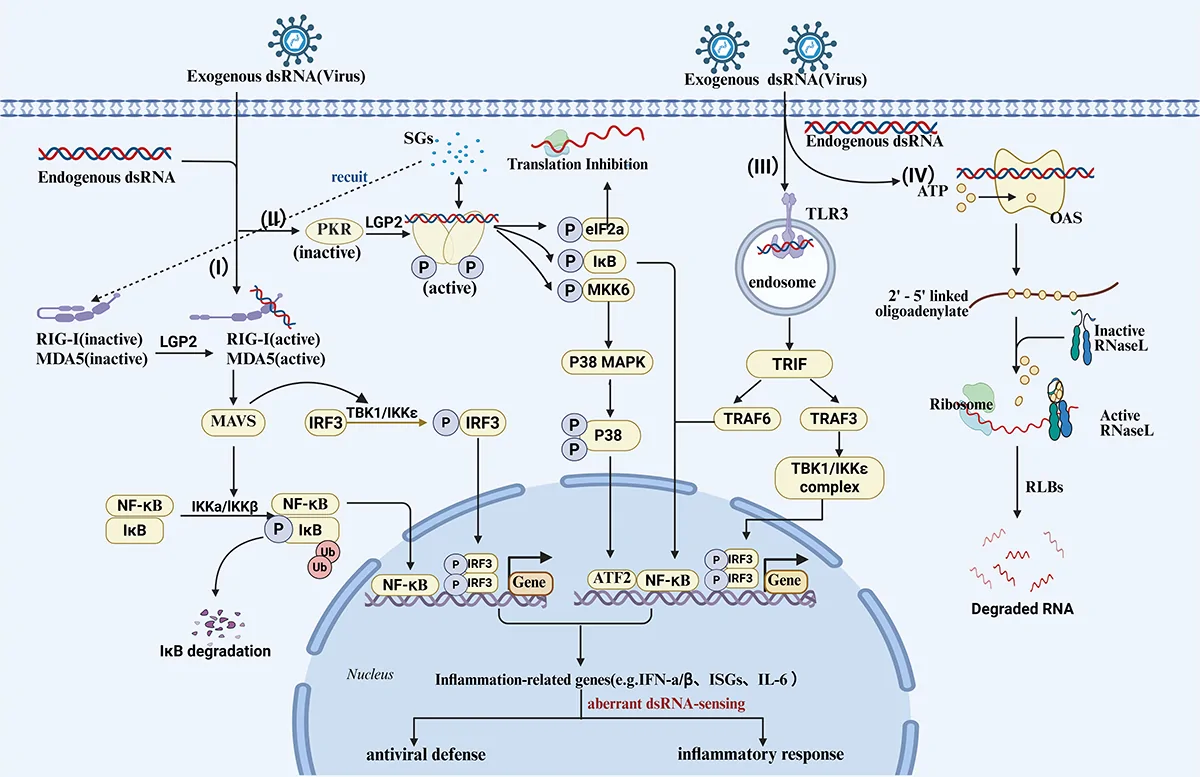
A multilayered and cross-amplified dsRNA immune sensing network based on RLRs, PKR, TLR3 and OAS. Cytosolic and endosomal dsRNA sensors cooperatively regulate interferon production, inflammatory gene expression, translational inhibition, and RNA degradation through interconnected signalling pathways, forming a multilayered antiviral immune network. Created in https://BioRender.com.

TLR3 is the only member of the human TLR family specialized for the recognition of long dsRNA, typically exceeding 40 bp in length. It predominantly localizes within endosomes. Although TLR3 expression has also been detected on the plasma membrane of certain cell types, such as mast cells [65], whether membrane-associated TLR3 is functionally competent for dsRNA sensing and signalling remains unresolved. Within the acidic endosomal environment, the extracellular domain (ECD) of TLR3 binds dsRNA and undergoes ligand-induced dimerization, triggering conformational changes in its Toll/IL-1 receptor (TIR) domain [66]. Activated TLR3 subsequently recruits the adaptor protein TIR-domain-containing adapter-inducing interferon-β (TRIF). TRIF initiates downstream signalling through both the TNF receptor-associated factor 6 (TRAF6)–NF-κB pathway and the TNF receptor-associated factor 3 (TRAF3)–IRF3 pathway. These signalling cascades ultimately induce transcription of IFN-β together with a range of pro-inflammatory cytokines, such as interleukin-6 (IL-6), interleukin-2 (IL-2) and tumour necrosis factor α (TNF-α) (Figure 3), thereby promoting antiviral immune responses [67].

When RIG-I and MDA5 misrecognize endogenous dsRNA that is inadequately edited by ADAR1 as non-self RNA [68,69], pathological activation of IFN-I signalling can occur, contributing to type I interferonopathies, most notably Aicardi–Goutières syndrome and Singleton–Merten syndrome [4,5]. Because RLR-mediated discrimination of dsRNA relies on subtle structural differences between viral and self RNA, maintaining a balance between antiviral defence and the prevention of inappropriate self-recognition remains a central challenge in understanding the pathogenesis of interferon-related disorders. In contrast, insufficient or dysregulated TLR3 activation may be associated with persistent viral infections [70].

#### 4.2.2. dsRNA-activated PKR signalling and translational reprogramming

PKR predominantly exists as an inactive monomer in the cytoplasm. It recognizes and binds dsRNA at least 30 bp in length through two tandem dsRNA-binding domains (dsRBD1 and dsRBD2). This interaction promotes PKR dimerization through its kinase domain, followed by autophosphorylation at Thr446 and Thr451 [37]. Assisted by the α-helix within dsRBD2, PKR undergoes conformational rearrangement, ultimately enabling full activation of its kinase activity [71]. Activated PKR phosphorylates eukaryotic initiation factor 2α (eIF2α), resulting in inhibition of translation initiation and consequent extensive translational repression (Figure 3). This process effectively inhibits viral protein synthesis and gene expression [72]. Against this backdrop of widespread translational suppression, a subset of host mRNAs undergoes selective translational activation. A hallmark of this phenomenon occurs during the integrated stress response (ISR), in which translation of activating transcription factor 4 (ATF4) is paradoxically enhanced. This event triggers the eIF2α-ATF4-CHOP (C/EBP homologous protein) axis, which promotes pro-apoptotic gene expression and cellular stress adaptation, thereby limiting viral dissemination [72]. This suggests that the PKR pathway does not merely suppress global translation; rather, it selectively reprograms translational output by inhibiting viral protein synthesis while prioritizing the translation of genes involved in cellular defence and repair. Beyond its role in translational control, PKR also functions as a signalling hub. It can phosphorylate the inhibitor of nuclear factor κB (IκB) and mitogen-activated protein kinase 6 (MKK6), thereby activating the NF-κB and p38 mitogen-activated protein kinase (p38 MAPK) pathways. This signalling cascade induces transcription of a broad array of inflammatory genes and promotes the production of pro-inflammatory cytokines (e.g. IFN-β, IL-6) (Figure 3), ultimately enhancing immune activation [73,74].

Notably, certain viruses have evolved mechanisms to counteract PKR activation. In some cases, viruses can even exploit activated PKR to facilitate their own replication, as demonstrated in lymphocytic choriomeningitis virus (LCMV) [75].

#### 4.2.3. dsRNA-mediated RNA degradation through OAS-RNase L axis

The human OAS family exhibits dsRNA length-dependent binding, with OAS3 showing a particularly strong preference for long dsRNA (>60 bp). OAS3 serves as a major dsRNA sensor responsible for activating the ribonuclease L (RNase L) pathway [76,77]. Upon binding dsRNA, OAS enzymes catalyse the polymerization of ATP into 2′–5′-linked oligoadenylates (2–5A). Acting as a second messenger, 2–5A promotes the dimerization and activation of RNase L. Once activated, RNase L mediates widespread degradation of intracellular RNA, leading to translational arrest and suppression of viral replication [78] (Figure 3). Furthermore, activation of RNase L induces the formation of distinct ribonucleoprotein structures known as RNase L–dependent bodies (RLBs) [79]. These structures facilitate the sequestration of subgenomic flaviviral RNA (sfRNA), preventing its interaction with P-body components and thereby enhancing RNA degradation efficiency [80].

However, Dengue virus (DENV) and Zika virus (ZIKV) can evade RNase L–mediated RNA degradation [81]. In response to such viral countermeasures, RNase L–mediated inhibition of mRNA nuclear export has emerged as a pivotal mechanism that broadly restricts both host and viral gene expression programmes [82].

#### 4.2.4. Integrated dsRNA sensing across the RLR, TLR3, PKR, and OAS pathways

The innate immune response triggered by dsRNA is not mediated by a single receptor but rather by an integrated sensing network characterized by spatial compartmentalization, functional redundancy, and synergistic amplification involving the RLR, TLR3, PKR, and OAS pathways (Figure 3). The OAS–RNase L axis represents a rapid first-line antiviral pathway that functions in a temporally complementary manner to other dsRNA sensors. Interferon regulatory factor 2 (IRF2) maintains basal OAS3 mRNA expression in unstressed cells [83], thereby enabling rapid RNase L activation during the early stages of infection and establishing a temporal window for the subsequent activation of RLR and PKR pathways. Following viral infection, IFN-β produced through the RLRs and TLR3 pathways cooperates with IRF2 to promote the phosphorylation of signal transducer and activator of transcription 2 (STAT2), leading to the induction of ISGs, including OAS3. This process establishes a positive-feedback loop within the RLRs/TLR3–OAS axis [84].

The RLR and TLR3 pathways exhibit spatial complementarity in dsRNA sensing. Their distinct localization within the cytosol and endosomes, respectively, enables comprehensive spatiotemporal surveillance throughout the viral life cycle, from initial entry to active replication. Functionally, these two pathways signal through MAVS and TRIF, respectively, converging on activation of IRF3 and NF-κB signalling cascades [61]. This coordinated signalling response synergistically induces the expression of IFN-β and ISGs, thereby ensuring robust antiviral responses and reducing the likelihood of immune evasion resulting from impairment of any individual pathway.

PKR acts as a vital signalling integrator within the RLR/TLR3–IFN-β axis. During hepatitis C virus (HCV) infection, PKR engages the key signalling components MAVS and TRAF3 through its dsRBDs, thereby facilitating IFN-β production [85]. Furthermore, PKR activation triggers the formation of stress granules (SGs), which serve as scaffolding platforms for the recruitment and enrichment of PKR and RLR components. This spatial organization further amplifies RLR-mediated signalling [86].

In summary, the RLR, PKR, TLR3, and OAS pathways do not function as isolated signalling modules. Rather, they are interconnected through extensive signalling crosstalk, functional redundancy, and cooperative amplification, forming a multilayered dsRNA sensing network that ensures robust and reliable detection of viral dsRNA while coordinating downstream antiviral gene expression programs. However, dsRNA sensing is not solely protective and requires tight regulation, as aberrant accumulation of endogenous dsRNA can trigger inappropriate immune activation, persistent interferon responses, and inflammatory disorders. In addition, it is worth noting that the genomic sources of endogenous dsRNA differ substantially across species, as exemplified by the Alu elements in primates and B1/B2 SINEs in rodents discussed above. This species specificity of endogenous dsRNA substrates raises an important question: Do different mammals rely on conserved dsRNA-sensing and surveillance mechanisms to regulate their distinct endogenous dsRNA repertoires? Cross-species complementation studies have shown that mouse ADAR1 p150 and MDA5 can functionally replace their corresponding human proteins [9], supporting functional conservation of the regulatory relationship between ADAR1-mediated editing of endogenous dsRNA and MDA5 surveillance in mammals. However, whether other dsRNA-sensing mechanisms, including TLR3, PKR, and OAS, exhibit similar cross-species functional conservation in responding to species-specific endogenous dsRNA remains unclear and requires further systematic investigation.

#### 4.2.5. Liquid–liquid phase separation in dsRNA-responsive gene regulation

Emerging evidence indicates that dsRNA sensors can undergo liquid-liquid phase separation (LLPS) to form dynamic biomolecular condensates [87,88]. These condensates facilitate rapid responses to dsRNA stimulation by concentrating signalling components while excluding unrelated molecules. For example, as a triggering signal, dsRNA binds to RLRs and subsequently drives LLPS of the downstream adaptor MAVS. Electron microscopy observations have revealed that MAVS condensates undergo a dynamic transition from liquid-like droplets to highly stable fibrillar aggregates. These MAVS condensates efficiently recruit key signalling molecules, including IRF3 and TBK1, thereby facilitating IRF3 phosphorylation and activating IFN-β transcription [89]. Another representative example involves PKR. dsRNA serves as a molecular scaffold that promotes the assembly of PKR into novel cytoplasmic condensates known as dsRNA-induced foci (dRIFs). These dRIFs selectively recruit antiviral proteins such as ADAR1 and DExH-box helicase 9 (DHX9), thereby lowering the activation threshold of PKR and amplifying the antiviral signal [90]. However, another view suggests that the formation of PKR-related condensates does not necessarily enhance downstream signalling. Zappa and colleagues reported that PKR forms another type of dynamic cluster, sequestering active PKR dimers from the cytoplasm and excluding its substrate eIF2α, thus buffering downstream signals and preventing excessive PKR pathway activation [91]. These findings suggest that certain condensates may function as signalling buffers rather than simply amplifying stress responses. Whether these observations reflect differences in condensate composition, maturation state, or experimental context remains unresolved. Thus, merely observing the formation of condensates is insufficient to predict signalling outcomes, and further studies are required to understand how different condensate states regulate PKR activity.

### 4.3. Non-canonical dsRNA-binding proteins in dsRNA-mediated gene regulation

Beyond its role in innate signalling pathways, dsRNA also regulates gene expression through interactions with non-canonical dsRBPs. These proteins are not primarily involved in innate immune signal transduction; instead, they play critical roles in controlling RNA fate and shaping cellular gene expression programmes. In this section, we focus on several representative dsRBPs that, upon binding to dsRNA, regulate gene silencing, maintain RNA homoeostasis, and modulate host RNAi-mediated RNA silencing. Representative examples and their biological functions are summarized in Table 1.

**Table 1.** Summary of gene expression regulatory mechanisms mediated by non-canonical dsRNA-binding proteins.

dsRBPs Name	Source	Structural Features	Recognition Mechanism	Functional Category	Ref.
Vigilin	Host	14 tandem KH domains	Recognizes inosine-containing dsRNA (I-dsRNA)	Transcriptional gene silencing	[92]
Staufen (STAU1/2)	Host	Multiple dsRBDs	Recognizes dsRNA in 3‘UTRs or complementary Alu elements (mRNA/lncRNA)	Post-transcriptional gene silencing	[93]
DHX9	Host	Two tandem dsRBDs + DExH motif helicase domain	Recognizes endogenous dsRNA structures (e.g. IR-Alus)	Maintenance of RNA homeostasis and gene expression stability	[94]
XRN1	Host	5‘→3’ exonuclease domain	Recognizes unprocessed endogenous dsRNA	Maintenance of RNA homeostasis and gene expression stability	[95]
NoV B2	Virus	Two positively charged, antiparallel α-helices as a homodimer	Recognizes long dsRNA	Antagonizing RNAi-mediated gene silencing	[96]
IAV NS1	Virus	Two positively charged, antiparallel α-helices as a homodimer	Recognizes long dsRNA	Antagonizing RNAi-mediated gene silencing	[96]
EV-A71 3A	Virus	Two positively charged, antiparallel α-helices as a homodimer	Recognizes long dsRNA	Antagonizing RNAi-mediated gene silencing	[96]
DENV2 NS2A	Virus	Positively charged residues (such as K135)	Recognizes long dsRNA	Antagonizing RNAi-mediated gene silencing	[96]
DXV VP3	Virus	Cation-rich region	Charge-dependent binding to long dsRNA	Antagonizing RNAi-mediated gene silencing	[97]
IIV6 340 R	Virus	dsRBD	Recognizes duplex siRNA	Antagonizing RNAi-mediated gene silencing	[98]
RuV capsid protein	Virus	Dimerization of the RuV capsid and the R169 and D171 residues	Recognizes duplex siRNA	Antagonizing RNAi-mediated gene silencing	[99]

#### 4.3.1. Gene silencing mediated by dsRNA–dsRBPs interactions

Mammalian Vigilin, also called high-density lipoprotein binding protein (HDLBP), is an RNA-binding protein containing 14 tandem KH domains that enable the specific recognition of inosine-containing dsRNA (I-dsRNA) generated by ADAR1-mediated A-to-I editing. Upon binding I-dsRNA, Vigilin forms a multiprotein complex with ADAR1, DHX9, and cofactors such as Ku70/80, thereby promoting activation of DNA-dependent protein kinase (DNA-PK). Activated DNA-PK subsequently phosphorylates the downstream effector KRAB-associated protein 1 (KAP1), which in turn recruits histone methyltransferases, including SETDB1 and suppressor of variegation 3–9 homolog 1 (SUV39H1). This recruitment results in the accumulation of repressive histone marks, including H3K9me3 and H3K27me3, at target chromatin loci. Ultimately, these epigenetic remodelling events prevent the recruitment of RNA Pol II, thereby achieving transcriptional gene silencing [92].

Staufen proteins (STAU1/2) recognize and bind dsRNA structures located within mRNA 3′ UTRs, thereby initiating Staufen-mediated mRNA decay (SMD). Target recognition primarily involves either intramolecular dsRNA structures within the mRNA itself or intermolecular dsRNA generated through base pairing between Alu elements in the mRNA 3′ UTR and complementary Alu-rich lncRNA or mRNA [93,100]. Upon binding these dsRNA regions, STAU1/2 recruit and promote the phosphorylation of UPF1, an ATP-dependent RNA helicase. Activated UPF1 exhibits enhanced ATPase and helicase activities, leading to mRNA decapping and subsequent degradation by 5′→3′ exonucleases and ultimately resulting in post-transcriptional gene silencing [101].

#### 4.3.2. Maintenance of cellular dsRNA homoeostasis by dsRNA–dsRBPs interactions

RNA homoeostasis encompasses not only the balance of RNA abundance but also the dynamic regulation of intracellular dsRNA levels. RNA helicase A (DHX9), a prototypical dsRBP within the helicase superfamily, utilizes its tandem dsRBDs and cooperates with ADAR1 to recognize dsRNA structures derived from endogenous retroelements, such as IR-Alus. Through its ATP-dependent DExH-box helicase activity, DHX9 unwinds dsRNA, thereby restricting aberrant dsRNA accumulation and maintaining intracellular dsRNA homoeostasis [57]. In the absence of DHX9, intracellular dsRNA accumulates markedly, leading to robust upregulation of immune-related genes, including transcription factor ATF3, pro-inflammatory cytokines such as IL-6 and IL-2, as well as proto-oncogenes JUN and FOS. Ultimately, this disruption of dsRNA homoeostasis results in increased cytotoxicity and cell death [94]. These findings suggest that disruption of dsRNA homoeostasis can trigger deleterious inflammatory responses and compromise cellular viability. Notably, DHX9 depletion has been shown to induce cytoplasmic dsRNA accumulation, thereby activating tumour-intrinsic innate immunity. These findings highlight DHX9 as a potential therapeutic target for enhancing antitumor immune responses [102].

In addition to dsRNA helicases, the RNA decay factor 5’−3’ exoribonuclease 1 (XRN1) contributes to dsRNA surveillance by degrading dsRNA derived from endogenous retroelements through its 5′→3′ exoribonuclease activity, thereby preventing aberrant dsRNA accumulation and preserving cellular homoeostasis. When XRN1 function is impaired, dsRNA homoeostasis is disrupted, leading to aberrant activation of dsRNA sensing pathways such as RIG-I and PKR, and sustained induction of ISGs [95]. Taken together, DHX9 and XRN1 function as critical guardians of intracellular dsRNA homoeostasis. Beyond preventing excessive dsRNA accumulation and inappropriate innate immune activation, they preserve RNA homoeostasis and contribute to the stability of global gene expression programmes.

#### 4.3.3. Viral dsRBPs as suppressors of RNAi-mediated gene silencing

In addition to host-encoded dsRBPs, many viruses encode dsRNA-binding proteins known as viral suppressors of RNAi (VSRs), which can antagonize host RNAi-mediated gene silencing. At the upstream stage of the RNAi pathway, several mammalian VSRs have been experimentally validated [96], including Nodavirus B2 protein (NoV B2), influenza A virus nonstructural protein 1 (IAV NS1), enterovirus A71 nonstructural protein 3A (EV-A71 3A), and dengue virus 2 nonstructural protein 2A (DENV2 NS2A). These proteins bind long dsRNA and block its processing by Dicer, thereby preventing siRNA biogenesis and enabling viral escape from RNAi-mediated silencing. Similarly, VSRs such as Drosophila X virus VP3 (DXV VP3) also exhibit viral suppressor of RNAi activity through dsRNA binding [97]. At downstream stages of the RNAi pathway, certain VSRs, including invertebrate iridescence virus-6 340 R (IIV6 340 R) [98] and rubella virus (RuV) capsid protein [99], suppress RNAi activity by binding siRNA duplexes and preventing their loading into the RISC, thereby impairing target viral RNA cleavage. Through structure-dependent recognition of dsRNA or siRNA at multiple stages of the RNAi pathway, these viral dsRBPs establish a viral RNA silencing antagonism mechanism. Therefore, viral dsRBPs provide important evidence for understanding the long-standing evolutionary competition between viral RNAs and the host RNAi silencing mechanism.

However, the biological significance of these viral antagonistic mechanisms varies significantly among different species. In plants, arthropods, and nematodes, cellular antiviral defence mainly relies on RNAi, and VSRs have evolved as important factors for viruses to antagonize this pathway. In contrast, some studies have indicated that the contribution of RNAi to antiviral defence in mammals is relatively limited. The interferon-mediated innate immune pathway activated by dsRNA, including the RLR, PKR, and OAS–RNase L signalling axes, is generally considered the main antiviral defence mechanism [96]. Therefore, during mammalian viral infections, whether VSRs mainly promote viral immune escape by antagonizing host RNAi-mediated gene silencing and what the actual contribution of this mechanism to viral replication and host defence is still require further investigation.

### 4.4. RNA–RNA duplex-associated mechanisms in gene expression regulation

Beyond canonical dsRNA-dependent pathways, local RNA–RNA duplexes formed through complementary pairing between regulatory ncRNAs and their targets can also contribute to gene expression regulation.

#### 4.4.1. lncRNA/circRN–miRNA–mRNA regulatory networks in post-transcriptional regulation

Competing endogenous RNA (ceRNA) networks represent one of the most extensively studied mechanisms of post-transcriptional gene regulation involving local RNA–RNA base pairing between miRNAs and their target transcripts. The core principle of this mechanism lies in the ability of different lncRNAs and circRNAs to competitively bind shared miRNAs through miRNA response elements (MREs). By acting as molecular sponges, these ncRNAs reduce the pool of free miRNAs. As a result, miRNA-mediated inhibition of target mRNAs, either through translational repression mediated by 3′ UTR binding or AGO2-dependent mRNA degradation, is attenuated [103] (Figure 4(A)). Within ceRNA networks, a single lncRNA or circRNA can interact with multiple miRNAs, thereby modulating miRNA availability and ultimately influencing their regulatory effects on target mRNA [104].

**Figure 4.**
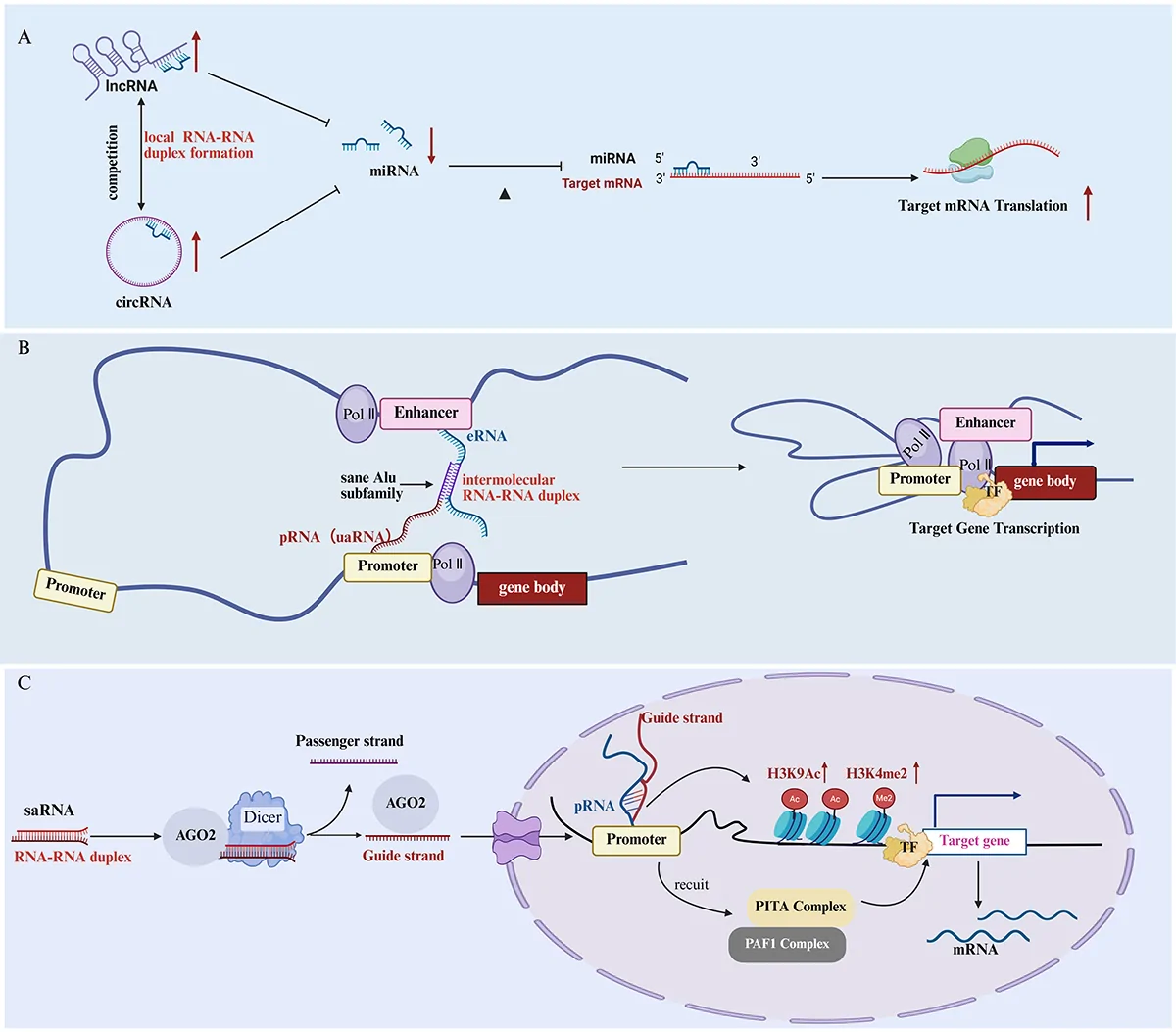
Non-coding RNA-mediated gene expression regulation through RNA duplex-associated mechanisms. (A) ceRNA network-mediated post-transcriptional regulation. (B) eRNA–pRNA duplex formation in enhancer–promoter communication and long-range transcriptional regulation. (C) RNA activation mediated by saRNA-directed promoter targeting. Created in https://BioRender.com.

The interactions within ceRNA networks are not unidirectional. On the one hand, lncRNAs that bind miRNAs can themselves become targets of the miRNA-AGO2 complex and undergo deadenylation, decapping, and exonuclease-mediated RNA degradation, thereby establishing a bidirectional regulatory relationship [105]. On the other hand, highly complementary ncRNA–miRNA pairing can trigger target-directed miRNA degradation (TDMD). This process involves ZSWIM8 E3 ubiquitin ligase complex-mediated degradation of AGO proteins, ultimately leading to miRNA destabilization. These findings indicate that ncRNA–miRNA interactions extend beyond competitive sequestration and actively regulate RNA turnover dynamics [106]. Furthermore, a subset of lncRNAs can bypass the canonical ceRNA mechanism by directly forming RNA–RNA duplexes with the 3’ UTRs of target mRNAs, thereby interfering with miRNA-mediated gene regulation. For instance, the lncRNA PXN–AS1 can form a stable RNA duplex with the 3’ UTR of PXN mRNA, thereby blocking miR-24-mediated mRNA degradation and consequently upregulating PXN mRNA expression [107]. This site-specific masking mode further expands the regulatory repertoire of RNA–RNA duplex-mediated post-transcriptional gene regulation.

However, whether ceRNA-mediated regulation represents a widespread physiological mechanism remains a matter of debate. Although numerous studies have reported that lncRNA and circRNA can regulate the expression of target genes by competitively binding to miRNA, quantitative analyses indicate that the ceRNA effect is strongly constrained by miRNA abundance, binding affinity, target-site abundance, and the stoichiometric relationship among ceRNAs, miRNAs, and their target transcripts. Denzler and colleagues found that the contribution of a single endogenous transcript to the total effective target abundance of miRNA is extremely low [108]. Therefore, under most physiological conditions, changes in a single ceRNA molecule are unlikely to significantly alter miRNA activity. However, this regulatory mode may become more prominent in pathological contexts such as cancer, where abnormally high expression of lncRNA or circRNA, or transcriptome remodelling, may enhance the ceRNA effect by increasing the abundance of miRNA-binding sites. In summary, canonical ceRNA regulation depends on base pairing between miRNAs and miRNA response elements on different target RNAs, while further quantitative and physiological studies are needed to rigorously validate predicted ceRNA networks.

#### 4.4.2. Enhancer RNA-promoter RNA duplexes in long-range transcriptional regulation

Recent studies have demonstrated that enhancer RNA (eRNA) can regulate promoter activity and promote target gene expression through the formation of RNA–RNA duplexes with promoter-associated RNA (pRNA). These eRNA-pRNA interactions are facilitated by the dimerization of RNA-binding proteins, which function as molecular bridges to establish an ‘RNA-protein-RNA’ complex [109]. This arrangement promotes chromatin looping and facilitates the spatial recruitment of transcriptional machinery to specific gene loci, thereby enabling long-range transcriptional regulation. In addition, eRNA and pRNA, particularly upstream antisense RNA (uaRNA), can directly form RNA-RNA duplexes through complementary sequences, especially Alu elements (Figure 4(B)). Such base-pairing provides molecular specificity for enhancer–promoter recognition. Experimental evidence has shown that deletion of a single Alu sequence from either the enhancer or promoter RNA significantly weakens long-range enhancer-promoter interactions and reduces target gene transcription [110,111]. These findings demonstrate that RNA duplexes formed through eRNA–uaRNA pairing are not merely a transcriptional byproduct but actively participate in long-range cis-regulation processes. This mechanism provides new insight into how RNA-RNA duplex formation contributes to the spatial organization and precise control of gene expression.

#### 4.4.3. saRNA-mediated RNA activation through promoter targeting

RNA activation (RNAa) is a biological phenomenon in which small activating RNAs (saRNAs), typically 19-nt duplexes with 2-nt 3’ overhangs, target gene promoters to induce transcriptional upregulation (Figure 4(C)). RNAa exhibits stringent target sequence requirements. Specifically, saRNAs generally avoid CpG islands and Alu elements, and their activity depends on both saRNA 5′-end modifications and the integrity and conservation of the target promoter sequences [112].

The core mechanism of RNAa is initiated by the loading of saRNA onto AGO2, facilitated by dsRNA-loading factors, followed by removal of the passenger strand to generate the guide strand-AGO2 complex [113]. Following nuclear translocation, this complex utilizes the seed region of the guide strand (2–8 nt) to specifically recognize target gene promoter or promoter-associated ncRNA through sequence complementarity. This recognition event subsequently promotes the recruitment of transcriptional activators and associated regulatory factors, thereby initiating gene activation [113]. This targeting process is accompanied by epigenetic remodelling at the promoter region, characterized by a reduction in repressive histone marks (e.g. H3K9me2) and enrichment of activating histone marks (e.g. H3K4me2, H3K9ac). These chromatin changes enhance chromatin accessibility, expose the transcription start site (TSS), and promote the recruitment of RNA Pol II and associated factors to the preinitiation complex, thereby driving robust transcriptional activation [114].

During this process, auxiliary factors such as the RNA polymerase II-associated factor 1 (PAF1) complex and the RNA-induced transcriptional activation (RITA) complex cooperate to facilitate RNAa-mediated chromatin remodelling and transcriptional elongation [115,116] (Figure 4(C)). Together with ceRNA networks and eRNA–pRNA interactions, RNAa illustrates the remarkable versatility of RNA-RNA duplexes in regulating gene expression across multiple regulatory layers, highlighting a unique mechanism of RNA-duplex-associated positive gene regulation.

### 4.5. Functional convergence of R-loop-derived RNA–DNA hybrids with dsRNA-mediated innate immune signalling

R-loops are a type of triple-stranded nucleic acid structure in eukaryotic cells, consisting of an RNA-DNA hybrid and a displaced single-stranded DNA strand [117]. They are not dsRNAs, and their recognition and metabolism involve partly distinct protein systems. R-loops are discussed here because R-loop-derived RNA–DNA hybrid duplexes can functionally converge with dsRNA-mediated innate immune pathways under specific conditions.

Beyond canonical dsRNA ligands, R-loops can activate innate immune signalling through the generation of immunostimulatory RNA–DNA hybrids. When the DNA damage repair factor breast cancer type 1 susceptibility protein (BRCA1) is dysfunctional or the RNA–DNA helicase senataxin (SETX) is depleted, transcription-associated R-loop resolution is impaired, leading to their abnormal accumulation. These accumulated R-loops can be processed by structure-specific endonucleases such as xeroderma pigmentosum complementation group F (XPF) and xeroderma pigmentosum complementation group G (XPG), generating RNA–DNA hybrids that are released into the cytoplasm. However, it is still unclear how R-loops are precisely regulated to be recognized and cleaved by XPF/XPG, and it is also unclear how these hybrids leave the nucleus. After entering the cytoplasm, these hybrids act as novel immunogenic species and can be recognized by the endosomal dsRNA sensor TLR3 and the cytosolic DNA sensor cyclic GMP-AMP synthase (cGAS). This recognition promotes IRF3 phosphorylation and induces IFN-β and ISG expression, thereby triggering innate immune responses [118,119] (Figure 5). Although TLR3 is classically regarded as a dsRNA sensor, the ability of RNA–DNA hybrids to activate TLR3 signalling suggests these structures possess dsRNA-like immunostimulatory properties. Therefore, although aberrantly processed R-loop-derived products do not represent canonical dsRNA structures, they may functionally converge with dsRNA-mediated innate immune pathways by engaging partially overlapping innate immune sensing mechanisms and inducing immune-related gene expression. Recent studies have further highlighted the importance of RNA structural homoeostasis in regulating such immune activation. Under transcriptional stress, MYC protein promotes the recruitment of the nuclear RNA exosome to regions enriched in R-loops and dsRNA-related structures, where the exosome degrades abnormally accumulated immunostimulatory RNA. When the RNA-binding region III (RBRIII) of MYC loses its function, it impairs exosome recruitment, leading to increased accumulation of R-loops and dsRNA and activating the TLR3-TBK1 immune pathway in cancer cells [120]. This study further revealed that RBRIII-mutant MYC retains the ability to restrict dsRNA loading onto TLR3 but fails to suppress RNA–DNA hybrid-mediated TLR3 activation [120]. This indicates that although dsRNA and RNA–DNA hybrids can functionally converge on the TLR3 signalling pathway, their different structures may be regulated by different upstream mechanisms. In addition, during abnormal R-loop accumulation and subsequent innate immune activation, the effects on disease outcomes may vary depending on the cellular environment. For example, in patient-derived cells from individuals with the neurodegenerative disorder ataxia with oculomotor apraxia type 2 (AOA2), this mechanism may contribute to cell death, thereby exacerbating neuronal loss. In contrast, during early tumorigenesis, this mechanism may have a protective role by eliminating genomically unstable cells in the context of BRCA1 mutations [119]. Therefore, whether the immune signals induced by R-loops promote disease development or act as a protective barrier remains an important unsolved question requiring further research.

**Figure 5.**
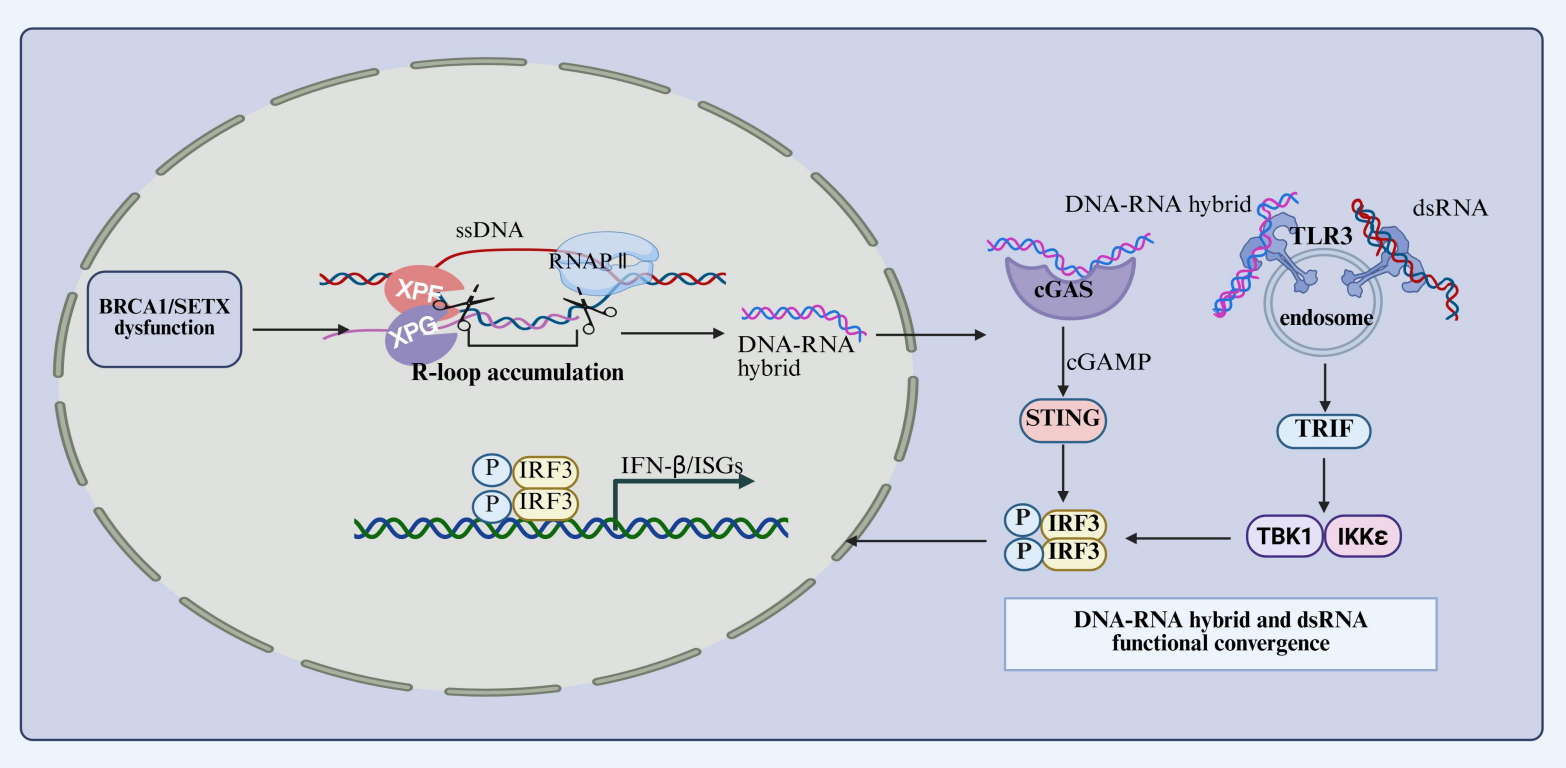
Functional convergence of R-loop-derived RNA–DNA hybrids with dsRNA-mediated innate immune signalling. R-loop-derived RNA–DNA hybrids can activate TLR3- and cGAS-mediated signalling, leading to IRF3 activation and IFN-I responses. Created in https://BioRender.com.

## 5. dsRNA in disease pathogenesis and progression

Across different disease contexts, dysregulation of dsRNA can occur at multiple stages of its cellular lifecycle, including dsRNA production, editing, immune sensing, and clearance. These alterations can affect the abundance, structural properties, or intracellular distribution of endogenous dsRNA, as well as the response thresholds of dsRNA sensors, thereby leading to aberrant activation of dsRNA-responsive pathways, including RLR–MAVS, PKR–eIF2α, and OAS–RNase L signalling. The resulting changes in interferon signalling, protein translation, RNA degradation, and cell fate provide a mechanistic link between dsRNA-mediated regulation and disease pathogenesis and progression.

### 5.1. dsRNA and cancer

Within the tumour microenvironment, cancer cells frequently undergo cancer-associated epigenetic reprogramming, such as widespread DNA hypomethylation and loss of heterochromatin. These changes can relieve epigenetic repression of TEs and promote the generation of endogenous dsRNA [121]. Aberrantly accumulated dsRNA can be recognized as a viral infection-associated nucleic acid signal, thereby triggering a viral mimicry response. Specifically, dsRNA can be sensed by receptors such as RLRs and TLR3, inducing IFN-I responses and widespread transcription of ISGs. In parallel, activation of the PKR and OAS–RNase L pathways suppresses global protein translation and promotes RNA degradation in tumour cells, respectively; when sustained or excessive, these responses can further promote tumour cell death [72,78,121]. Moreover, viral mimicry can induce immunogenic cell death (ICD), resulting in the release of tumour antigens and DAMPs. This process enhances antigen presentation by dendritic cells and promotes CD8^+^ T-cell activation, thereby translating tumour-intrinsic dsRNA responses into adaptive antitumor immunity [122].

Conversely, tumour cells have evolved multiple strategies to counteract viral mimicry and achieve immune evasion. These strategies include downregulation of key signalling molecules such as MAVS [123], ADAR1-mediated hyper-editing of inverted repeat elements (IREs), and altered processing of ISG mRNAs [124]. Additionally, during tumour progression, both IFN-β induced by RLR activation and IFN-γ secreted by activated T cells and NK cells can upregulate the expression of programmed death-ligand 1 (PD-L1). This feedback loop contributes to the establishment of an immunosuppressive tumour microenvironment [125], thereby further facilitating immune escape. More recently, studies have shown that tumour cells can suppress viral mimicry by limiting the cytoplasmic export of dsRNA precursors. Retroelement-derived nuclear RNA transcripts require the mRNA export machinery to reach the cytoplasm, where they can subsequently form dsRNA and activate antitumor immunity. However, tumour cells can aberrantly regulate coactivator-associated arginine methyltransferase 1 (CARM1) to enhance RNA exosome activity while suppressing the expression of RNA export factors (e.g. DDX39A and ALYREF). This process reduces cytoplasmic dsRNA accumulation and attenuates immune activation [126]. These findings suggest that dsRNA-mediated viral mimicry is tightly regulated by nuclear–cytoplasmic RNA transport pathways.

### 5.2. dsRNA and autoimmune diseases

Aberrant recognition of endogenous dsRNA by innate immune sensors can disrupt immune homoeostasis and drive persistent activation of IFN-I signalling. ADAR1 deficiency and gain-of-function (GOF) mutations in IFIH1, which encodes MDA5, disrupt self-dsRNA tolerance at distinct regulatory levels. ADAR1 deficiency does not necessarily increase the total abundance of dsRNA; rather, insufficient A-to-I editing renders endogenous dsRNA more immunostimulatory and more readily recognized by MDA5. In contrast, IFIH1 GOF mutations enhance the responsiveness of MDA5 to endogenous dsRNA, effectively lowering the threshold for self-RNA recognition [127]. Despite these mechanistic differences, both defects converge on aberrant activation of the MDA5–MAVS signalling axis, resulting in sustained IFN-I production and ISG expression. Persistent activation of this pathway disrupts immune tolerance and contributes to the development of type I interferonopathies [127]. Aicardi–Goutières syndrome (AGS) represents a prototypical example of these rare interferonopathies and is associated with ADAR1 loss-of-function (LOF) or IFIH1 GOF mutations. Genetic evidence further indicates that these mutations are often mutually exclusive in patients with AGS. Although these mutations affect different steps of dsRNA regulation, both ultimately promote aberrant MDA5 activation and sustained IFN-I signalling [4,5]. Beyond AGS, other disorders, including Singleton–Merten syndrome, as well as more prevalent autoimmune diseases such as psoriasis, systemic lupus erythematosus (SLE), and rheumatoid arthritis (RA), have also been associated with dysregulated dsRNA homoeostasis or sensing [128].

### 5.3. dsRNA and neurodegenerative diseases

The nervous system is particularly sensitive to perturbations in RNA homoeostasis, partly because neurons maintain unusually high basal levels of endogenous dsRNA. Neuron-specific expression of the ELAVL family proteins markedly extends the 3′ UTRs of mRNAs, thereby increasing dsRNA formation and accumulation, a phenomenon that is even more pronounced in ADAR1 knockout cells [129]. This elevated basal dsRNA level has been proposed to represent a pre-adapted antiviral state, enabling neurons to rapidly respond to viral infection. However, it also renders neurons particularly susceptible to pathological dsRNA accumulation. Under conditions such as ADAR1 deficiency, excessive dsRNA accumulation can trigger pathological inflammation and neuronal death, contributing to the development of multiple neurodegenerative disorders. In patients with amyotrophic lateral sclerosis (ALS) and frontotemporal dementia (FTD), accumulated neuronal dsRNA has been shown to induce IFN-I responses, promote neuroinflammation, and drive neuronal death [130]. Moreover, animal studies have linked dsRNA accumulation to loss of function of the RNA-binding protein TDP-43 [131]. In addition, increased levels of dsRNA and MDA5, together with evidence of enhanced pro-inflammatory signalling, have been observed in brain tissues from patients with Alzheimer’s disease (AD) and progressive supranuclear palsy (PSP) [132]. These findings suggest that dsRNA-mediated immune activation may represent a shared contributor to neuroinflammatory pathology in several neurodegenerative diseases.

## 6. Therapeutic potential of dsRNA-based strategies

### 6.1. dsRNA-based gene silencing therapy through RNAi

dsRNA-mediated gene silencing through RNAi has become one of the cornerstone strategies in modern nucleic acid therapeutics. Synthetic siRNAs are loaded into AGO2-containing RISC complexes, where the guide strand directs sequence-specific recognition and degradation of complementary target mRNAs [133]. This mechanism enables selective suppression of disease-associated genes and has become one of the most clinically advanced applications of dsRNA-mediated gene regulation. In 2018, patisiran, the first siRNA drug, received U.S. Food and Drug Administration (FDA) approval for the treatment of hereditary transthyretin-mediated amyloidosis (hATTR) [134]. Subsequently, the second-generation hATTR therapy vutrisiran was developed and successfully translated into clinical practice [135]. Since then, several liver-targeted siRNA therapeutics have gained regulatory approval. For example, lumasiran is used for the treatment of primary hyperoxaluria type 1 (PH1). By silencing the hepatic metabolic enzyme HAO1, it reduces urinary oxalate levels, enabling most patients to achieve normal or near-normal urinary oxalate within six months of treatment [136]. Similarly, inclisiran lowers low-density lipoprotein cholesterol (LDL-C) levels by silencing the hepatic PCSK9 gene, providing sustained lipid lowering with only twice-yearly maintenance dosing compared with conventional statin therapy [137]. However, currently approved siRNA therapeutics are predominantly restricted to liver-targeted applications. The development of efficient and tissue-specific delivery systems for extrahepatic targets therefore remains a major bottleneck to the broader clinical application of dsRNA-based therapies.

### 6.2. dsRNA-based gene activation therapy through RNAa

In contrast to RNAi-mediated gene silencing, therapeutic RNAa exploits promoter-targeted saRNA mechanisms described in section 4.4.3 to induce sequence-specific transcriptional activation, offering new opportunities for the treatment of cancer and other genetic disorders. For example, saRNA targeting the promoter of vitamin D_3_ up-regulated protein 1 (VDUP1) can induce VDUP1 transcription, thereby significantly inhibiting lung cancer cell proliferation [138]. RNAa-based strategies have also shown therapeutic potential in the treatment of rare diseases. For instance, saRNA-mediated activation of the muscleblind-like protein 1 (MBNL1) has been shown to alleviate aberrant splicing events associated with myotonic dystrophy [139]. Despite these promising advances, RNAa-based therapeutics remain at an early stage of development. Efficient and tissue-specific delivery of saRNA to diseased cells continues to represent a major obstacle to clinical translation.

### 6.3. dsRNA-based viral mimicry therapy

dsRNA-based viral mimicry therapies aim to increase the production or accumulation of immunostimulatory dsRNA, thereby activating dsRNA-sensing pathways that are frequently attenuated in tumour cells [121]. Several therapeutic strategies have been developed to exploit the viral mimicry properties of dsRNA. For instance, the epigenetic agents azacitidine and decitabine, which induce endogenous dsRNA production through the activation of TEs, have been approved by the U.S. FDA for the treatment of haematological malignancies, such as myelodysplastic syndromes (MDS) and chronic myelomonocytic leukaemia (CMML). These agents promote viral mimicry by reducing DNA methylation, thereby increasing dsRNA levels and activating RLRs and TLR3-mediated IFN-I signalling, along with other dsRNA-responsive pathways. In this way, dysregulated dsRNA homoeostasis is converted into an antitumor immune signal [140]. Furthermore, a fixed-dose combination of decitabine and cedazuridine, developed to improve oral bioavailability, has been approved in the United States and Canada for the treatment of MDS and CMML [141]. In contrast to epigenetic agents that increase endogenous dsRNA, synthetic dsRNA analogs such as poly(I:C) and its derivative poly-ICLC directly activate dsRNA sensors, including TLR3 and MDA5, thereby enhancing vaccine immunogenicity [142]. For example, in a phase I clinical trial of a melanoma vaccine, poly-ICLC was used as a central adjuvant in combination with other immunostimulatory agents, and the combination significantly enhanced vaccine immunogenicity [142,143]. However, the immunostimulatory activity of dsRNA must be precisely controlled to avoid immune tolerance or excessive immune activation. Achieving this balance requires optimization of dsRNA dosage, delivery platforms, and T-cell functional states [144]. Furthermore, studies have shown that combining dsRNA-based viral mimicry therapy with epigenetic therapy and immunotherapy can achieve superior antitumor efficacy [145]. For example, in a phase II clinical trial in ovarian cancer, the combination of guadecitabine (a DNMT inhibitor) and pembrolizumab (an anti–PD-1 antibody) achieved durable clinical benefit in 31.4% of patients [146].

### 6.4. Control of dsRNA byproducts in mRNA vaccine development

The development of in vitro transcribed (IVT) mRNA vaccines for infectious diseases and cancer was rapidly accelerated during the COVID-19 pandemic. However, the IVT process inevitably generates dsRNA byproducts. These dsRNA molecules are recognized by cellular sensors, triggering the expression of IFN-I and ISGs. This recognition not only elicits unintended immune and inflammatory responses but also induces translational inhibition through the PKR-eIF2α signalling axis, thereby reducing the expression of the vaccine antigen encoded by the target mRNA [147]. Consequently, stringent control of dsRNA content is paramount to ensuring the safety and efficacy of mRNA vaccines. Current removal strategies include high-efficiency purification techniques, such as reversed-phase high-performance liquid chromatography (RP-HPLC) and cellulose-based chromatography, which effectively enrich the target single-stranded mRNA while eliminating dsRNA contaminants [148]. In addition, optimization of IVT reaction conditions, such as the use of high-temperature IVT protocols or DNA templates containing encoded poly(A) tails, can reduce the generation of dsRNA byproducts at the source [149].

## 7. Conclusions and perspectives

Over the past decade, our understanding of dsRNA has undergone a profound paradigm shift, evolving from its traditional perception as a mere marker of viral infection to its recognition as a central regulatory molecule operating in both nuclear and cytoplasmic compartments. dsRNA not only mediates gene silencing through cytoplasmic and nuclear RNAi pathways but also functions as a central driver of innate immunity by orchestrating IFN-I production, immune-related gene transcription, and translational repression through dsRNA sensors. Furthermore, intricate interactions between dsRNA and diverse classes of non-canonical immune dsRBPs, together with local RNA–RNA duplexes formed through complementary ncRNA pairing, contribute to the regulation of transcription, epigenetic states, and RNA homoeostasis. The functional convergence of R-loop-derived RNA–DNA hybrids with dsRNA-responsive pathways, despite their structural distinction from dsRNA, has further broadened the conceptual framework of dsRNA-associated regulation. Importantly, dysregulation of these processes is closely associated with the initiation and progression of a wide spectrum of diseases, including cancer, autoimmune disorders, and neurodegenerative pathologies.

Despite these advances, our understanding of dsRNA remains incomplete in terms of both gene expression regulation mechanisms and disease pathogenesis and progression. First, endogenous dsRNA exhibits substantial heterogeneity in length, terminal architecture, 5′-end processing, and chemical modifications, while its spatial and temporal dynamics remain poorly characterized. Consequently, the principles governing the sorting of distinct dsRNA species into specific regulatory pathways remain elusive. Second, the structural distinctions between host-derived and viral dsRNA are often remarkably subtle. This creates a fundamental biological challenge: aberrant recognition may trigger immunopathologies such as interferonopathies, whereas excessive suppression can compromise antiviral immunity. Maintaining the balance between self-tolerance and effective antiviral defence therefore remains a major challenge in the field. Third, the broader physiological relevance of some dsRNA- and RNA duplex-associated regulatory mechanisms remains uncertain, as current evidence is often derived from specific experimental or pathological contexts. Fourth, dsRNA-sensing networks comprise multilayered regulatory factors and intricate feedback loops. How these pathways are coordinated by different cellular regulators and how their dysregulation contributes to disease remain poorly understood. Finally, although dsRNA-based therapies are advancing rapidly, critical challenges such as targeted delivery, molecular stability, immunogenicity control, and minimization of off-target effects must be overcome before their full clinical potential can be realized.

## Data Availability

No data was used for the research described in the article.
